# Origin of the Avalanche-Like Photoluminescence from Metallic Nanowires

**DOI:** 10.1038/srep18857

**Published:** 2016-01-05

**Authors:** Zongwei Ma, Ying Yu, Shaoxin Shen, Hongwei Dai, Linhua Yao, Yibo Han, Xia Wang, Jun-Bo Han, Liang Li

**Affiliations:** 1 Wuhan National High Magnetic Field Center and School of Physics, Huazhong University of Science and Technology, Wuhan 430074, China; 2 School of Physics, Peking University, Beijing 100871, China; 3 Department of Physics, Xiamen University, Xiamen 361005, China; 4 Wenhua College, Wuhan 430074, China

## Abstract

Surface plasmonic systems provide extremely efficient ways to modulate light-matter interaction in photon emission, light harvesting, energy conversion and transferring, etc. Various surface plasmon enhanced luminescent behaviors have been observed and investigated in these systems. But the origin of an avalanche-like photoluminescence, which was firstly reported in 2007 from Au and subsequently from Ag nanowire arrays/monomers, is still not clear. Here we show, based on systematic investigations including the excitation power/time related photoluminescent measurements as well as calculations, that this avalanche-like photoluminescence is in fact a result of surface plasmon assisted thermal radiation. Nearly all of the related observations could be perfectly interpreted with this concept. Our finding is crucial for understanding the surface plasmon mediated thermal and photoemission behaviors in plasmonic structures, which is of great importance in designing functional plasmonic devices.

Surface plasmonic systems have been extensively studied for decades due to their significant roles in revealing underlying mechanisms about light-matter interactions on nanoscale^1^,^2^,^3^,^4^, and extensive applications in areas such like subwavelength imaging^5^, photothermal therapy^6^,^7^,^8^, ultrasensitive biosensing^9^, data storage^10^, solar cells^3^, and photodetectors^11^, etc.

The excitation of surface plasmon resonances (SPR) gives metallic nanostructures excellent optical properties compared to bulky ones, such as surface plasmon resonance enhanced photoluminescence, local field enhanced second- and third-order optical nonlinearities, and surface plasmon enhanced light harvesting and biosensing, *et al.* On the one hand, SPR excitation enhances both the incoming and outgoing electromagnetic fields^12^,^13^. On the other hand, the strong localized fields greatly enhance the light absorption, especially multiphoton absorption, of incident light. Therefore, SPR enhanced multiphoton luminescence (MPL) from surface plasmonic systems has attracted much attention^12^,^14^,^15^,^16^,^17^,^18^. Generally, the MPL spectral intensity *I*_MPL_ is a power function of the excitation power density (*P*_EPD_), the slope of the dependent curve in a logarithmic coordinate (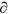
log*I*_MPL_ / 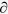
log*P*_EPD_) should be integers because of the quantum nature of light.

Most of the reported MPL in plasmonic systems involve slope values no more than four. However, an avalanche-like photoluminescence (APL) with slopes as large as 18.3 was observed in 2007 in coupled Au nanowires (NWs)^19^, and subsequently in Ag NW arrays^20^, Ag NW monomers^21^, and Au nanorod-nanoparticle hybrids^22^, respectively. In these works, measurements and calculations showed a typical bi-segmental EPD dependence of the APL intensity (*I*_APL_). A dependence of *I*_APL_ on excitation polarization, which was correlated to the field enhancement factor, was also observed. Besides, an ultraslow decay rate of the APL intensity obtained from lifetime measurements was quite different from other reported MPL observations^14^,^23^. The authors attributed the APL to strong longitudinal SPR coupling and cross-relaxation between nearby NWs, or radiative recombination of holes and electrons that were excited to high levels by multiphoton absorption and/or multiphoton excitations of surface plasmons. But the intrinsic mechanism is still not clear and the real origin of this APL behavior is still confusing, especially when a series of research results about ultrafast SPR and hot carrier relaxation processes are taken into consideration^1^,^4^. In this paper, we systematically investigated the APL behavior in a Au NW array from a new point of view that differs from the above ones. A reasonable explanation was proposed, expecting to put an end to this controversy about the origin of APL in metallic NWs. We will show that our explanation could lead to a better insight into the light-matter interaction on the nanoscale.

## Results and Discussion

**Figure 1:**
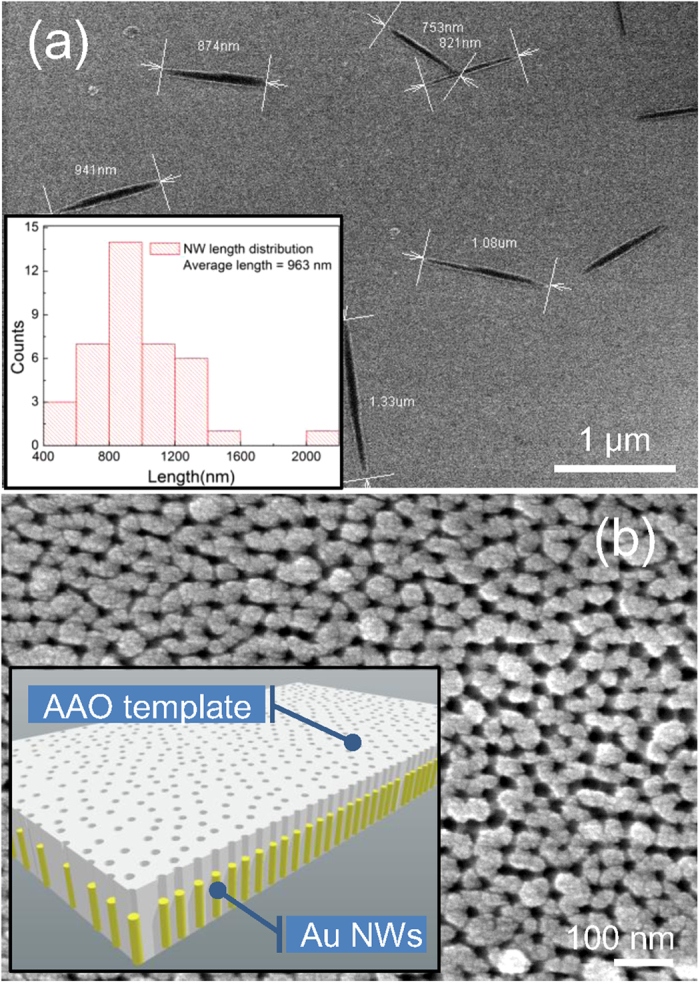
Sample characterization and structure schematic illustration. (**a**) SEM image of the Au NW monomers with the length distribution shown in the inset. The average length of the NWs is 963 nm. (**b**) Top view of the AAO template. Randomly distributed holes with average diameter and center-to-center distance of 20 nm and 45 nm, respectively, are clearly seen. Inset: schematic diagram of the sample.

We fabricated an Au NW array in a porous anodic aluminum oxide (AAO) template by electrochemical deposition method^24^. The NWs are aligned perpendicularly to the template surfaces and parallel to each other (see Figure S1 in supporting information). Figure 1a shows the scanning electron microscopy (SEM) image of the dispersive NW monomers released from the template. An average length about 963 nm is extracted from the length distribution analysis (inset). Figure 1b is the top view SEM image of the AAO template, indicating an average hole diameter of ~20 nm and center-to-center distance between adjacent holes of ~45 nm, respectively. The diameter of each NW is limited by the hole size where it was deposited in, however, the coating layer, which is inferred from energy dispersive X-ray spectroscopy (EDS) analysis (see Figure S1) to be mainly composed by Na_2_CO_3_, makes the NWs to look thicker and straight in the SEM images.

**Figure 2:**
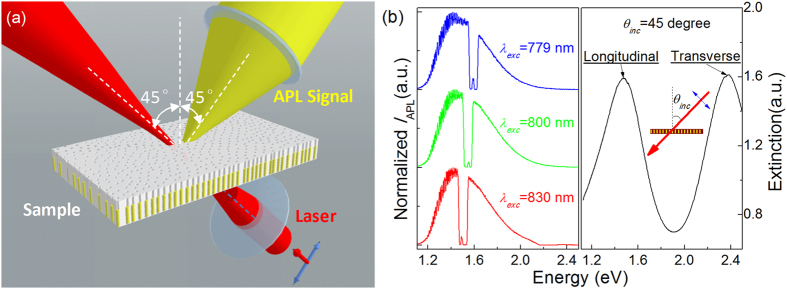
APL excitation configuration and APL/extinction spectra. (**a**) Light path configuration for sample excitation and APL signal collection. The focused Gaussian laser beam was p−polarized. (**b**) Left panel: APL spectra excited by laser of three different wavelengths. Hollows in the spectra are caused by notch filters. Right panel: Extinction spectra of the sample. Transverse and longitudinal SPR bands locate at 2.37 eV and 1.47 eV, respectively.

Typical APL spectra were obtained by exciting the sample with a polarized continuous wave (CW) laser beam at constant EPD. The APL signal was collected on the contrary side of the sample to minimize the noise from laser (see Fig. 2a and Figure S2 for more information about the experimental setup). Notch filters were utilized to block the scattered laser, which results in a great suppression of photocounts around excitation laser wavelength in the spectra. Wide-band continuum luminescent spectra taken under 779 nm, 800 nm, and 830 nm laser excitation show no distinct difference, as shown in left panel of Fig. 2b. This is because the radiation ability of each NW as a nanoantenna is proportional to the surface plasmon density of states (SP-DOS)^25^, which is an intrinsic quantity of the sample and just depends on the structural parameters^26^,^27^. The full width of half maximum (FWHM) of the spectrum is very large, for which at least three reasons should be considered: (1) the nonuniformity of NW lengths, (2) broadening effect of plasmon dephasing^1^, and (3) Plasmon hybridization from inter-rod coupling effect^28^,^29^. But the observation of APL in Ag NW monomers demonstrates that coupling between NWs is not the crucial reason for APL generation^21^, so the array should be treated as an anisotropic collection of NW monomers here. As for the profile of the luminescent spectra, their resemblance to SPR extinction spectra has been widely reported in some noble metal nanostructures^15^,^30^,^31^,^32^. However, this resemblance is broken in our case since an asymmetrical APL spectrum profile and a nearly symmetrical one of longitudinal SPR extinction band were both observed in the same nanostructure. The extinction spectrum, measured at a 45 degree incident angle and p−polarization, is shown in right panel of Fig. 2b for comparison, from which two SPR extinction bands locating at around 1.47 eV (longitudinal) and 2.37 eV (transverse) are clearly seen. Besides, no APL peak in the spectra corresponding to the transverse SPR extinction band was observed.

The APL signal exhibit similar excitation polarization dependence (see Figure S3) to those reported in Ref. 20, 21. Thus, to efficiently excite the longitudinal SPR mode of the array, we fixed the excitation polarization inside the incident plane (*p*−polarization) and the wavelength *λ*_exc_ around 800 nm.

**Figure 3:**
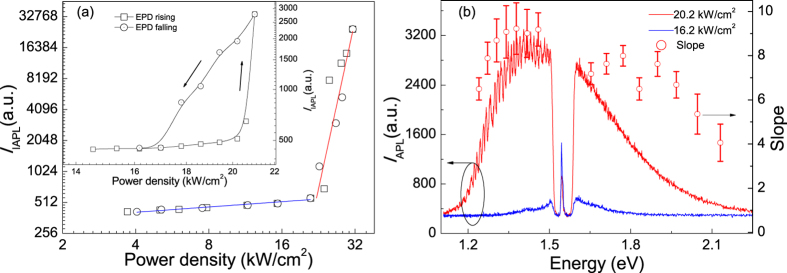
EPD dependence of integrated APL intensity/spectra and slope variation versus photon energy. (**a**) The EPD dependence of integrated APL intensity (^I^_IAPL_) appears as a typical bi-segmental curve in logarithm coordinate. *EPD*_th_ locates at around 20.2 kW/cm^2^ and the two slopes are fitted to be *b*_1_ = 0.17 ± 0.02 and *b*_2_ = 10.87 ± 1.74, respectively. The inset shows a fine structure of the data curve around *EPD*_th_. (**b**) PL spectra taken below (blue) and above (red) *EPD*_th_, respectively. Open circles with error bars represent the fitted slope (*b*_2_) values versus photon energy.

The EPD dependent APL was investigated and the results are presented in Fig. 3. The APL intensity was integrated over the “APL region” (i.e. spectral region outside the hollow) and plotted as a function of EPD in Fig. 3a. With the coordinate set into logarithm form, a typical bi-segmental APL curve is clearly seen. The APL threshold EPD (*EPD*_th_) locates at around 20.2 kW/cm^2^. Open squares and circles are data obtained from EPD rising and falling processes, respectively, which coincide well with each other in a wide EPD variation range. The solid lines in blue and red are fitting curves according to function *Y* = *ax*^*b*^, where *b* is the slope (*b* = 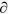
log*I*_MPL_ / 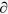
log*P*_EPD_) and the values of it below and above the threshold are fitted to be *b*_1_ = 0.17 ± 0.02 and *b*_2_ = 10.87 ± 1.74, respectively. These values, including the previously reported ones of APL, distribute randomly in a wide range rather than locate near integers. This suggests the APL may not be a strict MPL behavior. Inset of Fig. 3a is a fine structure of the EPD dependence of APL intensity near *EPD*_th_. It is clear that the *EPD*_th_ value in the rising EPD sweeping curve is higher than that in the falling one.

Moreover, instead of being unchanged, the slope *b*_2_ was found to vary with the emitted photon energy, as shown in Fig. 3b. The variation trend of it resembles the APL spectra profile. The deviation at higher energy side of the peak may be caused by a blueshift of the APL peak, as will be discussed later. These slope values are much larger than most of the reported ones in plasmonic systems, and their distribution also seems to be random. It further excludes the possibility that the APL behavior originates from MPL. Besides, the photoluminescence spectra below (blue) and above (red) *EPD*_th_ differ a lot, suggesting a possible transformation of the luminescent mechanism at *EPD*_th_.

**Figure 4:**
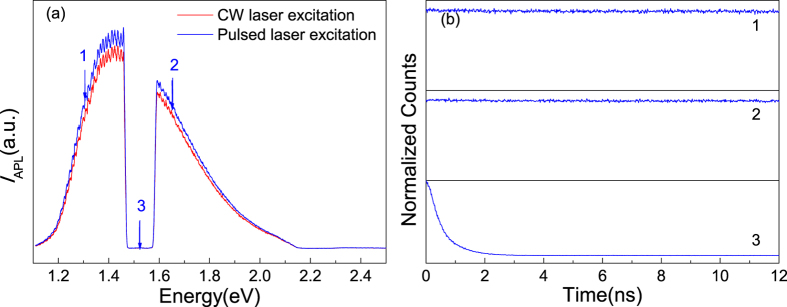
CW/pulsed laser excited APL spectra comparison and lifetime measurement results. (**a**) Comparison of APL spectra excited by CW and fs pulsed laser, respectively, with the mean EPD kept constant. (**b**) Lifetime measurement results of the APL signal (1 and 2) and scattered laser (3), the latter reflects the system response.

The lifetime of APL was measured by using a time correlated single photon counting (TCSPC) system with the sample excited by a pulsed laser (130 fs, 76 MHz). Before that, a comparison of the APL spectra excited by CW and pulsed laser beams at the same mean EPD was made, and no significant difference was found (Fig. 4a). In the following measurements, an extremely long lifetime of APL signal (far beyond measurable for our TCSPC system) was obtained at both sides (numbered 1 and 2) of the excitation laser wavelength (817 nm). The decay curve of the scattered laser (numbered 3), which is limited by the system response, is presented as reference (see Fig. 4b). The above facts demonstrate that, the long lifetime APL should not come from direct radiative damping of excited SPR which usually happens within 100 fs after excitation^1^,^4^,^23^,^32^,^33^,^34^,^35^,^36^,^37^,^38^. Although the generated hot carriers may transfer their energy to radiative photons via plasmon restimulation^32^, this process is still limited by the ultrashort hot carrier relaxation time which is on a scale of several picoseconds, far below expectation from our experimental results. Therefore, some energy-saving mechanism must be responsible for APL so that this ultra-slow behavior is only related to the mean EPD.

**Figure 5:**
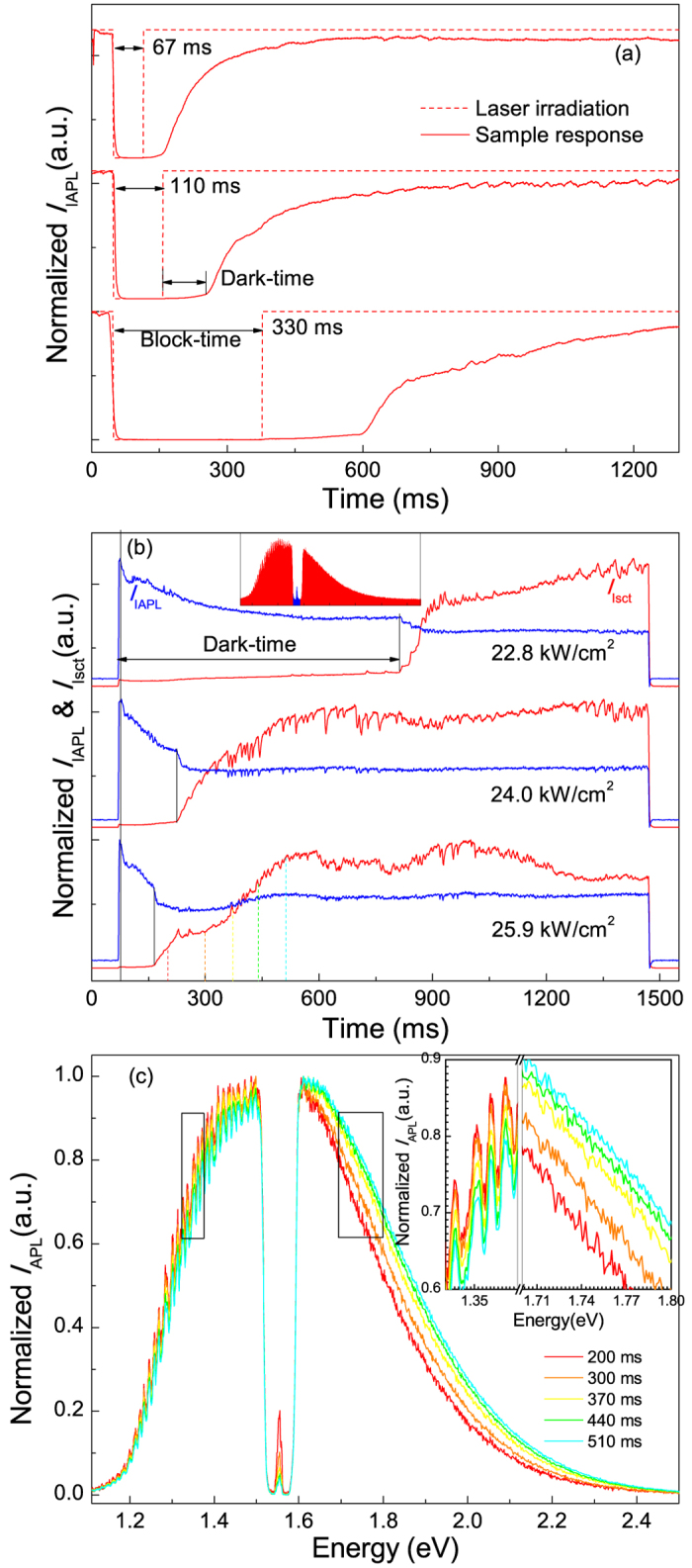
Dynamic properties of APL. (**a**) Dynamic response of APL intensity with CW laser irradiation being blocked for different periods of time. (**b**) Dynamic response of the APL and scattered laser intensities versus the CW laser EPDs. (**c**) Normalized APL spectra selected from the rising side of APL dynamic process in (**b**), indicating an obvious blueshift of the APL peak.

To find more clues, the dynamical properties of the APL behavior on millisecond (ms) timescale were studied by fast blocking the laser beam for different periods of time (block-time) and monitoring the APL recovery processes with a spectrometer after the sample was irradiated again. The EPD value was kept constant at 30.3 kW/cm^2^, which was above the threshold. The integrated APL curves for three typical block-times are shown in Fig. 5a, where the red dashed lines show the time of duration of laser irradiation. The APL intensity is found to experience a “dark-time” before recovering to original level. As the block-time increases, the dark-time gets longer.

Since Landau damping and the following accompanying hot carrier thermalization and relaxation processes would transfer the energy of plasmon resonance into the metal lattice as well as the AAO template in the form of heat in a quite short time no more than 10 ns^4^, the Au lattice temperature grows due to heat accumulation until the energy input and output get equivalent. When the incident laser beam is blocked, the lattice temperature drops. A larger temperature reduction during the block-time requires a longer time to recover the temperature, or the PL intensity. The APL is therefore inferred as a temperature related behavior. If this is true, then thermal radiation seems to be a proper candidate for APL origination^39^, because the independence of APL on material (Au/Ag), degree of aggregation (monomer/array) as well as excitation wavelength is consistent with Planck’s law of black-body radiation which tells us that the radiation ability of a perfect black-body at certain photon energy only depends on its temperature. Hence, the falling rate that is much larger than the rising one of APL intensity (see Fig. 5a) indicates a rather large heat conduction power compared to the radiation power. And it can be inferred that higher heating efficiency would be achieved if the localized field intensity inside the metallic NWs is stronger (or field enhancement factor is larger)^21^,^22^. More measurements and calculations were carried out to check the reasonability of the proposed thermal radiation model for APL origination.

Temperature is one of the most important quantities that determine the black-body radiation spectrum. So we examined the effect of different EPDs on the APL property by comparing the dynamical variation processes of the integrated APL intensity (*I*_IAPL_, red) and scattering laser intensity (*I*_Isct_, blue) in Fig. 5b. The spectral regions over which the integrals were performed are indicated in the inset by corresponding colors. Three features of the EPD dependent dynamical processes can be summarized from these results as follows: (1) both *I*_IAPL_ and *I*_ISCT_ show bi-segmental dynamical processes; (2) for each EPD value, *I*_IAPL_ and *I*_ISCT_ always transit from the first segments to the second ones synchronously in time sequence; (3) the first segments of both intensities, or namely the dark-time segments, shrink at higher EPDs. We will show that these features can be primely interpreted with the temperature dependent thermal radiation model.

*I*_Isct_ variation can be seen as a measure of the variation of energy conversion from laser to luminescence, so the two intensities act in an opposite manner. To better understand the plasmon induced thermal effect on APL, the *EPD*_th_ could be equally expressed as temperature threshold *T*_th_ at which APL occurs, even though it is hard to measure. When higher incident EPDs are applied, shorter time is needed for the lattice to be heated to *T*_th_. So the achieving of *T*_th_ puts an end to the dark-time and starts the avalanche-like process, resulting in bi-segmental response property in both dynamical process curves.

At microscopic scales, the black-body radiation described by Planck’s law originates from thermally excited propagating electromagnetic waves that leave the surface of thermal emitter in the form of photons^40^,^41^. Kirchhoff’s law connects the emissivity of a material directly to its absorptivity at equilibrium, so the radiation from a metallic nanostructure is tailored by its absorption property. In fact, many works have been done to engineer the thermal radiation or absorption properties by designing structures with specific SPR absorption spectra, or SP-DOS distribution^42^,^43^,^44^,^45^,^46^,^47^,^48^,^49^. Hence, the energy utilization efficiency could be improved by selective suppressing the unwanted thermal radiation^48^ or heat conduction. However, most of these works concentrated on devices working in infrared spectral region.

In our case, the NW array can be treated as a collection of nanoantennas for visible/near-infrared thermal radiation^50^. The large aspect ratio of the NWs makes them easier to be heated by laser^51^, and the small average separation between nearby NWs offers a highly efficient (far beyond the limitation of Planck’s law) near-field heat transferring channel by tunneling of thermally excited evanescent waves^2^,^41^,^52^,^53^,^54^,^55^. This tunneling effect not only holds back the fast increase of temperature, but also leads to quenching of photoemission at the existence of Al foil substrate as we have observed. At regular temperatures, hundreds of Kelvin for example, the dominant thermally excited electromagnetic wave energy lies far below the longitudinal SPR energy band, so the radiation is suppressed for a period of time (the dark-time). Meanwhile, the absorption of excitation energy by the array grows in the longitudinal SPR band as the temperature goes up, according to Kirchhoff’s law, resulting in a decrease of scattered laser intensity during the dark-time. When *T*_th_ is reached, the avalanche-like process begins as the thermally excited electromagnetic wave energy starts to markedly intersect with the SPR extinction band. Therefore, more heat energy is allowed to couple to longitudinal SPR and finally leave the sample through radiative decay of SPR, giving the APL spectra that we observed. A final temperature/APL intensity is reached once the input (laser radiation) and output (thermal radiation and heat conduction) power get equivalent. Since the absorption of laser is further enhanced in this process, we can see now that the shift of *EPD*_th_ shown in inset of Fig. 3a is caused by the difference in laser absorption before and after *T*_th_ is reached.

For black-body radiation, two significant features should be addressed. One is the great enhancement of radiation intensity (similar to our APL) with the growing of temperature^46^,^48^,^56^, the other one is a blue-shift in photoemission spectra according to Planck’s law. Fortunately, we did find both in our sample. As shown in Fig. 5c, some spectra were picked out from the rising side of the dynamical APL process when the EPD was 25.9 kW/cm^2^, as indicated by the dashed lines in different colors. Clear blue-shift can be seen in the normalized spectra. Hence, the APL is proven to be SPR assisted thermal radiation, which means, SPR excitation not only helps to heat the metal lattice by generating hot electrons, but also selectively couples thermal radiation into far field luminescence.

**Figure 6:**
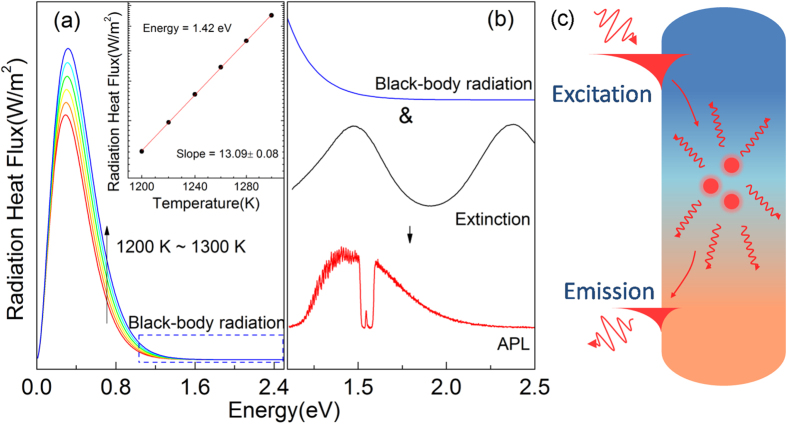
Black-body calculation and schematic illustration of the proposed model. (**a**) Black-body radiation calculation in the 1200K ∼ 1300 K temperature region according to Planck’s law. Inset:the variation of monochromic heat flux at 1.42 eV versus temperature gives a slope of 13.09 ± 0.08 in logarithmic coordinate. (**b**) The formation of the asymmetric APL peak is interpreted qualitatively as an extinction spectrum tailored monodecreasing black-body radiation curve in the corresponding spectral region. (**c**) The proposed model is illustrated to interpret the origin of the APL from metallic nanowires as an SPR assisted thermal radiation.

As evidence, we calculated black-body radiation spectra according to Planck’s law by estimating the Au lattice temperature in the range from 1200 K to 1300 K with an interval of 20 K. The estimation was made based on a slow decay of APL intensity during a long time observation under laser irradiation of 30.3 kW/cm^2^ (see Figure S4). Some of the NWs were believed to be deformed in this process, so *T*_th_ is estimated to lie below the melting point of the NWs^57^. Despite that the reported temperature rise in other plasmonic systems are not as large to give pronounced photoemission in the visible region, the relatively small heat capacity of porous AAO template and poor heat dissipation condition of air circumstance at room temperature in our case still makes it possible. Another proof is that, thinner AAO templates are more easily damaged by the large amount of heat generated by the NWs than thicker ones, so the local temperature must be extremely high. From the calculation, a series of monotonic decreasing curves in the spectral region that we are interested in (i.e. 1.1 ∼ 2.5 eV, Fig. 6a) were obtained. By plotting the calculated heat flux values at 1.42 eV versus temperature in a logarithmic coordinate system and linearly fitting the data points, we got a slope value of 13.09 ± 0.08 (see inset of Fig. 6a), which is on the same order of magnitude with the measured ones in this paper (9.01 ± 1.09, Fig. 3b) as well as other works^19^,^20^. Although this comparison is rough, it does help to understand the role played by black-body radiation in the APL behavior in nanostructures of this kind. In Fig. 6b we qualitatively explain the formation mechanism of an asymmetrical APL curve (or a tailored black-body radiation spectrum), by combining a monotonic decreasing black-body radiation curve together with a nearly symmetrical extinction one. The radiation in the transverse SPR band vanishes because no thermal radiation falls into it at this temperature range.

Figure 6c is a schematic illustration of our model that the APL is indeed a kind of SPR assisted thermal radiation in the visible region. The radiated energy comes from thermally excited electromagnetic waves that fall into the SPR band of the NW. APL only occurs when the metal lattice temperature is sufficiently high that these electromagnetic waves are efficiently coupled to SPR and finally radiated to the far field. It is an efficient way to heat the nanostructure by means of SPR resonant excitation with laser, since the enhanced absorption and localized field intensity improves the local energy density, and the large penetration depth of the incoming field involves more metal atoms in heating^51^. Moreover, the suppression of unwanted radiation and heat conduction also helps to minimize the waste of heat energy, which is beneficial for the increase of temperature of the metal lattice.

In summary, we have fabricated an Au NW array using electrochemical deposition method in a porous AAO template and investigated the APL behavior systematically under different excitation conditions. The results showed that both the APL intensity and the dynamical processes were sensitive to the mean EPD, but not dependent on the excitation wavelength. After excluding the possibility of APL originating from direct radiative decay of surface plasmon resonance or recombination of electron-hole pairs through the observation of extremely long APL lifetime, we proposed a new model, i.e. surface plasmon assisted thermal radiation, to explain the APL in Au and other metallic NWs, in which temperature was a key factor to control the APL behavior. The observed blue-shift of APL peak and the good agreement between numerical calculations and experimental observations confirmed the correctness of our explanation. These results and analysis not only provide new insights into the APL mechanism of metallic/plasmonic nanostructures, but also demonstrate that thermal radiation can be readily extended to visible region by designing plasmonic nanostructures appropriately, and exciting them with laser beams rather than complicated heating equipments. This APL behavior may find potential applications in bio-detectors and new photoemission devices.

## Methods

### Fabrication and characterization

The anodic aluminum oxide (AAO) template was fabricated by using a two-step anodization method^24^. A 99.99% aluminum sheet, electrochemically polished in ethanol solution of HClO_4_ (V_HClO4_ :V_ethanol_ = 1:3) at 16 V and 4 °C for 3.5 min with a Pt counter electrode, was used for first anodization in 0.3 M sulfuric acid solution at 19 V and 4 °C for 4.75 h, after which the aluminum oxide layer was removed by wet chemical etching in 60 °C mixed solution of chromic acid (wt 1.5%) and phosphoric acid (wt 6%) for 10 h. Then a second anodization process under exactly the same condition as former was applied for 13 h 40 min to get a new aluminum oxide layer thick enough to avoid being damaged by strong laser irradiation in the latter optical measurements. The ordered hole array was obtained by stepwise reducing the anode voltage to 6 V. The Au NW array was deposited into the porous AAO template by applying an alternating current (AC, 8 V, 50 Hz) in a mixture of HAuCl_4_ (10 mM) and H_2_SO_4_ (0.03 M) solution. Finally, the Al foil was removed with oversaturated solution of HgCl_2_.

NWs were released by dissolving the template with NaOH aqueous solution (1 M). The mixture was diluted with ethanol and then centrifuged for 6 min at a speed of 6000 r/min. Bottom layer of the solution was saved for morphology characterization. The morphology of the AAO template and NWs are characterized by scanning electron microscopy (SEM). The component of the NWs released from the template was determined by energy dispersive X-ray spectroscopy (EDS) analysis (See Figure S1 for more details).

### Optical measurements

The extinction measurement was performed with a UV-Vis-IR spectrophotometer (PerkinElmer, Lambda 950). In order to observe both the transverse and longitudinal SPR extinction bands, the sample was mounted on a homemade stage to make sure the incident angle was 45 degree, and besides, the incident laser beam is linearly polarized by a Glan-Tylor prism along the axis direction of the NWs (p−polarization).

Photoluminescence and lifetime measurements were also carried out at an incident angle of 45 degree. A Ti:Sapphire laser source (Coherent, Mira-900) working at continuous wave (CW) or pulsed (130 fs, 76 MHz) output modes served as the laser source. An attenuator and a half wave plate were used to modulate the excitation power and to adjust the laser polarization direction, respectively. The laser beam was focused by a convex lens (f = 75 mm) onto one side of the sample and the APL signal was collected from the other side of it (see Fig. 2a and Figure S2) to avoid disturbance of signal by reflected laser. A small portion (2:02%) of laser power was reflected by a glass slide into a power meter to monitor the EPD at the front surface of the sample under oblique incident condition. All EPD values in the text are calibrated. The APL and scattering signals were collected by a group of lenses into a spectrometer (Andor, SP-970) for spectral analysis. Notch filters centered at different wavelengths were used as laser blockers, so they may induce suppression of photon counts around excitation wavelength in the spectra. The lifetime measurements were performed taking advantage of a time correlated single photon counting (TCSPC) system.

In the dynamical process observations, a mechanical shutter was employed to modulate the CW laser irradiation into square wave pulses. The signal spectra were recorded by the CCD of the spectrometer at a frequency of 1000 Hz. Detailed system configuration can be found in supporting information (Figure S2).

### Theoretical calculation

Black-body heat flux calculation was done with Matlab R2014a according to Planck’s law of black-body radiation^39^:


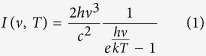


where *I* (*v, T*) is the heat flux radiated by a perfect black-body, *h* is the Planck constant, *c* is the speed of light in vacuum, *v* is the frequency of electromagnetic radiation, *k* is Boltzmann constant and *T* is the absolute temperature of the black-body. The temperature was estimated to locate below the melting point of Au NWs, so we set a series of temperature values from 1200 K to 1300 K with an interval of 20 K.

## Additional Information

**How to cite this article**: Ma, Z. *et al.* Origin of the Avalanche-Like Photoluminescence from Metallic Nanowires. *Sci. Rep.* **6**, 18857; doi: 10.1038/srep18857 (2016).

## Supplementary Material

Supplementary Information
