# Supplementary material for: Origin of the Avalanche-Like Photoluminescence from Metallic Nanowires
Source: Sci Rep. 2016 Jan 5;6:18857. doi: 10.1038/srep18857 (PMC4700426; doi:10.1038/srep18857)
Supplement: Supplementary Information [file srep18857-s1.pdf]

Supplementary Information

Origin of the Avalanche-like Photoluminescence from Metallic nanowires

Zongwei Ma,† Ying Yu,‡ Shaoxin Shen,¶ Hongwei Dai,† Linhua Yao,† Yibo Han,§ Xia Wang,‖ Junbo Han,*,§ and Liang Li§

†*Wuhan National High Magnetic Field Center and School of Physics, Huazhong University of Science and Technology, Wuhan 430074, China*

‡*School of Physics, Peking University, Beijing 100871, China*

¶*Department of Physics, Xiamen Unimersity, Xiamen 361005, China*

§*Wuhan National High Magnetic Field Center, Huazhong University of Science and Technology, Wuhan 430074, China*

‖*Wenhua College, Wuhan 430074, China*

E-mail: junbo.han@mail.hust.edu.cn

This document contains figures and discussion regarding sample characterization(analysis by scanning electron microscopy, SEM and energy dispersive spectrum, EDS), schematic diagram of experimental setup for avalanche-like photoluminescence(APL) investigation, excitation polarization dependence, and a slow decay curve of the APL intensity versus time.

Sample characterization

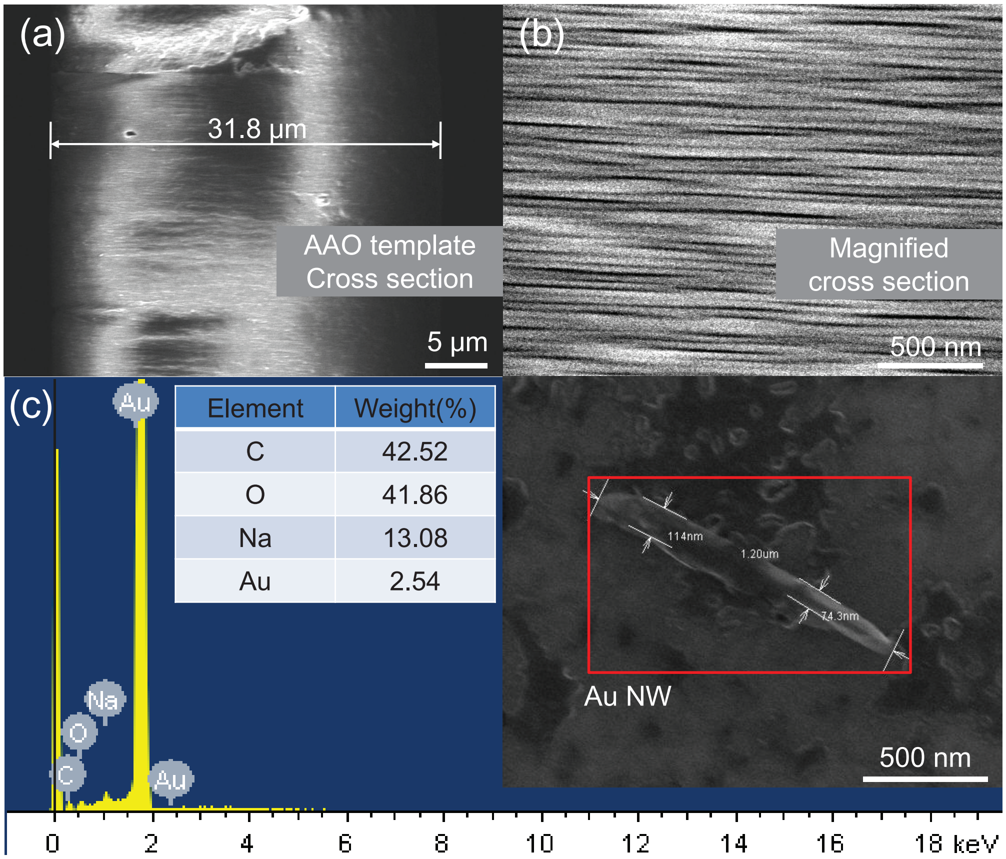

Figure S1: SEM and EDS characterization of the template and nanowire. (**a**)SEM image of the cross section of AAO template, showing the AAO template thickness $\sim$ 31.8 μm. (**b**)Magnified cross section image. (**c**)EDS analysis on the squared area of the inset.

SEM and EDS analysis were performed and the results are presented in Fig. S1. The thickness of the AAO template is about 31.8 μm. This large thickness prevents the sample from burning out easily. The magnified SEM image in Fig. S1b shows parallel holes in the cross section of the AAO template. Only a small portion of the atoms in the EDS analysed area in Fig. S1c is Au, suggesting a small diameter of the nanowire. The rest elements C, O, and Na belong to Na_2CO_3, *i.e.*, the reaction production of CO_2 and NaOH used for template dissolution. The nanowires are coated by Na_2CO_3, making them look thicker than bare ones.

Experimental setup

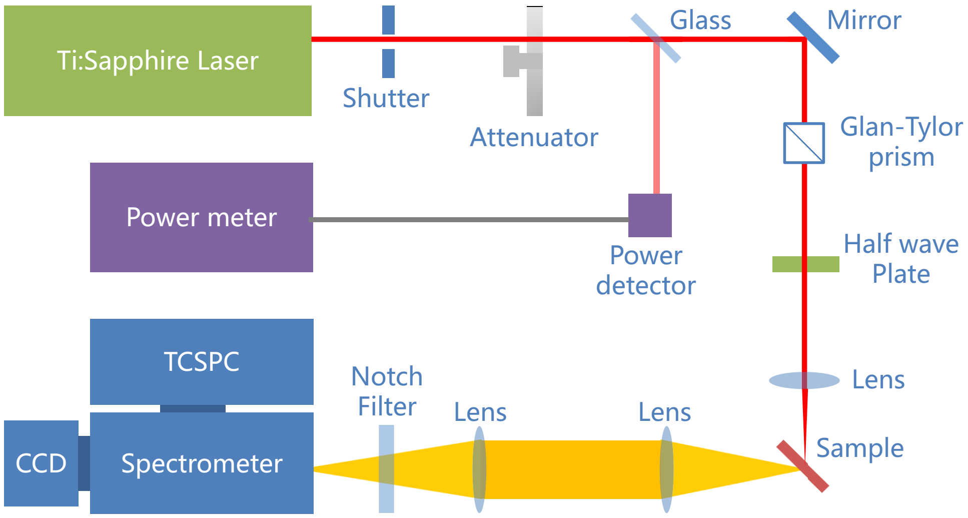

Figure S2: Experimental setup.

Excitation polarization dependence

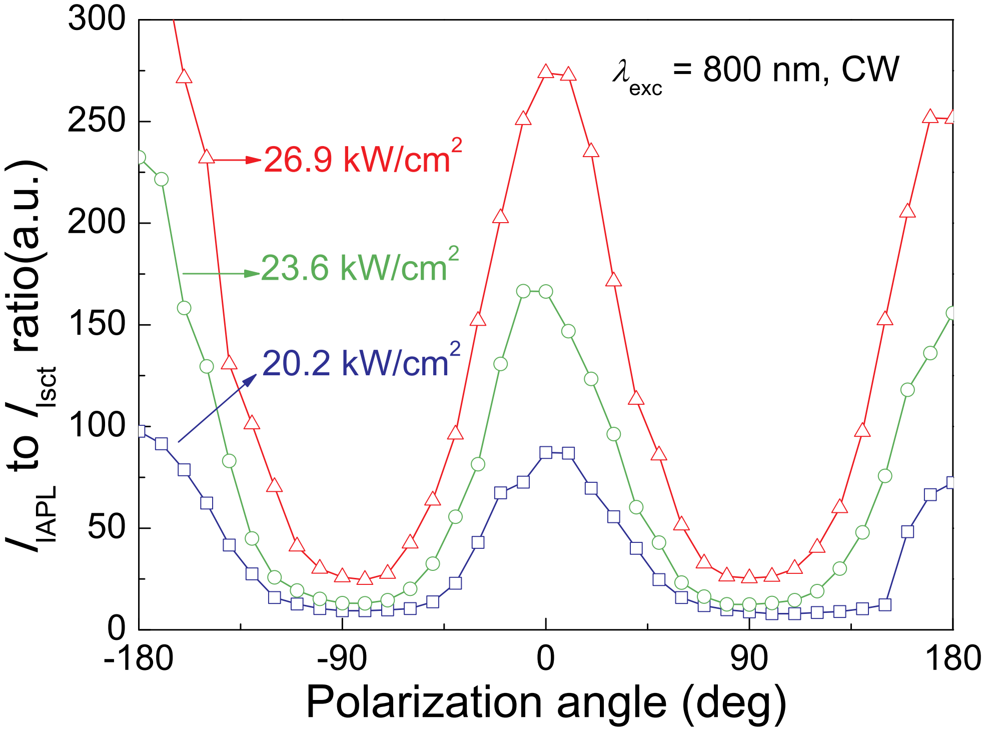

Figure S3: Excitation polarization dependence. The APL to scattered laser ratios(meant to minimize the influence of laser intensity fluctuation) under excitation of 800 nm CW laser beams of different power densities as a function of polarization angle are depicted. A typical dipolar behavior can be clearly seen from this result.

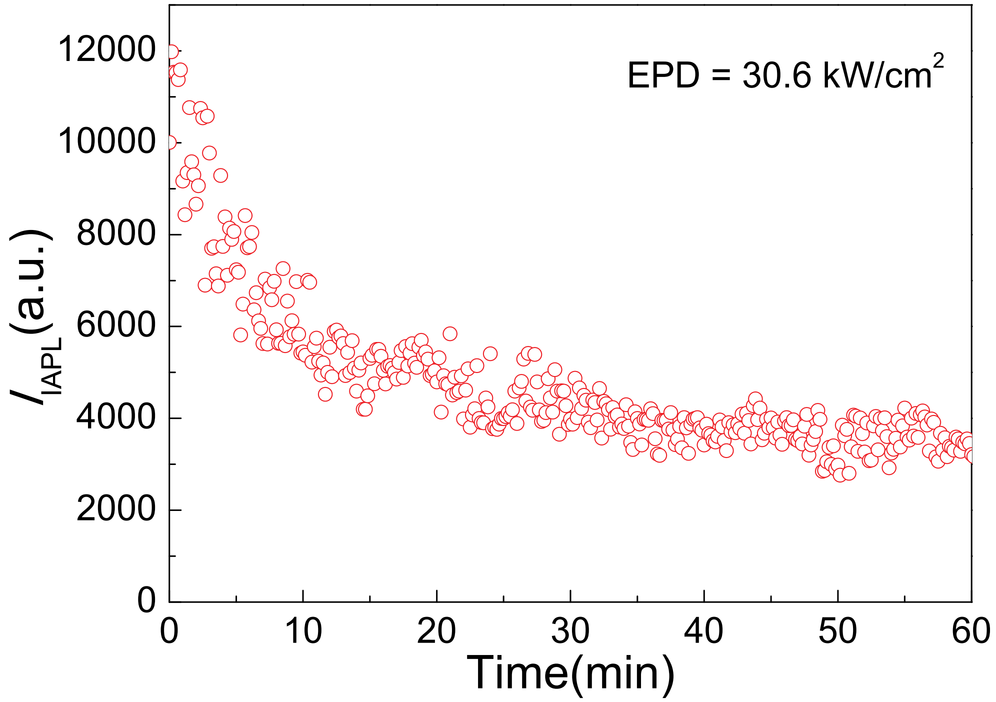

Figure S4: Ultraslow APL intensity decay process. A relatively high excitation power density of 30.6 kW/cm^2 was used.
